# Iron metabolism strategies in diatoms

**DOI:** 10.1093/jxb/eraa575

**Published:** 2021-03-06

**Authors:** Xia Gao, Chris Bowler, Elena Kazamia

**Affiliations:** 1 Institut de Biologie de l’ENS (IBENS), Département de Biologie, École Normale Supérieure, CNRS, INSERM, Université PSL, 75005 Paris, France; 2 John Innes Centre, UK

**Keywords:** Diatoms, ferritin, iron physiology, iron quotas, iron starvation-induced proteins (ISIPs), iron storage, iron uptake

## Abstract

Diatoms are one of the most successful group of photosynthetic eukaryotes in the contemporary ocean. They are ubiquitously distributed and are the most abundant primary producers in polar waters. Equally remarkable is their ability to tolerate iron deprivation and respond to periodic iron fertilization. Despite their relatively large cell sizes, diatoms tolerate iron limitation and frequently dominate iron-stimulated phytoplankton blooms, both natural and artificial. Here, we review the main iron use strategies of diatoms, including their ability to assimilate and store a range of iron sources, and the adaptations of their photosynthetic machinery and architecture to iron deprivation. Our synthesis relies on published literature and is complemented by a search of 82 diatom transcriptomes, including information collected from seven representatives of the most abundant diatom genera in the world’s oceans.

## Teaser

Diatoms are responsible for an estimated fifth of the total carbon fixation on Earth. Here we review their relationship with a key micronutrient in a changing ocean: iron.

## Introduction

Unicellular life began to evolve more than 3 billion years ago in an ocean that was devoid of molecular oxygen and rich in soluble ferrous iron (Fe^2+^). The metabolic processes that became core to all cellular life relied on the abundance of this transition element and its ability to stably occupy multiple valence states, as cofactor to enzymes catalysing reactions involving the transfer of electrons. Most notably, these processes include photosynthesis and respiration, as well as the synthesis of essential organic molecules such as amino acids, lipids, deoxyribonucleotides, and sterols. The minimum concentration of iron required by an individual cell to sustain its metabolic functions is referred to as its metabolic ‘iron quota’. Within cells, iron homeostasis is carefully controlled, since overabundance of Fe^2+^ can catalyse the formation of damaging reactive oxygen species. While the preference of cells for reduced iron has remained unchanged, that is, cells readily take up Fe^2+^ and carefully regulate its concentration intracellularly, the abiotic environment of unicellular species living in today’s ocean is vastly different from that existing at the start of these organisms’ evolution. The contemporary surface ocean is oxygenated and well mixed, with a mildly alkaline pH (global averages are around 8.2; Lauvset *et al.*, 2015). This is an oxidizing environment that chemically shifts iron into its ferric state (Fe^3+^), so that the most abundant form of iron is as Fe^3+^ bound by organic material or colloid particles of oxyhydroxides (Gledhill and Buck, 2012). Organisms have therefore had to evolve a variety of molecular mechanisms to make such recalcitrant forms of iron bioavailable.

**Fig. 1.**
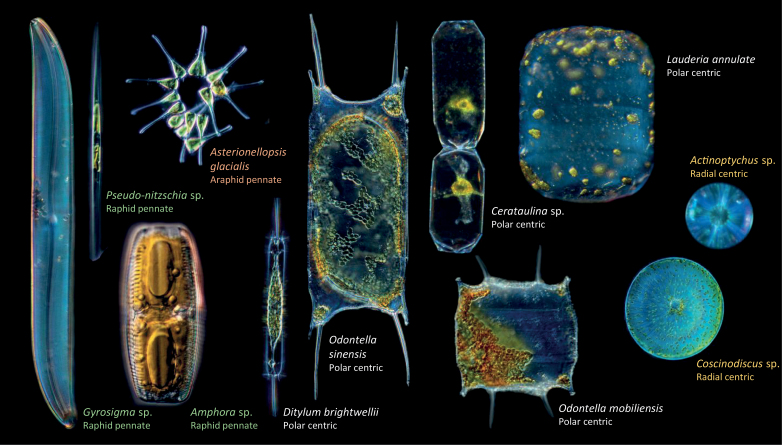
Light microscopy images of representative species in the raphid and araphid pennate, and polar and radial centric groups of diatoms. Sizes are not to scale. Diatoms were collected and photographed on board *Tara*, in Roscoff, Villefranche-sur-Mer Marine Stations (France), and Bigelow Laboratory, Boothbay (USA). Photography by Christian and Noé Sardet, rearranged from C. Sardet, 2015. Plankton: Wonders of the Drifting World. Republished with permission of University of Chicago Press; permission conveyed through Copyright Clearance Center, Inc.

One of the dominant, ubiquitous groups of photosynthetic producers in the contemporary ocean is diatoms, which are estimated to account for 20% of total global primary production (~20×10^15^ g carbon fixed per year) (Field *et al.*, 1998; Mann, 1999). Diatoms are unicellular stramenopiles (heterokont protists within the chromalveolates) and are, in an evolutionary context, a relatively young form of life. Molecular phylogenies date the origin of diatoms towards the beginning of the Mesozoic Era (Sims *et al.*, 2006), while current estimates of the richness of diatom species vary between 40 000 and 200 000; counterintuitively, the higher end of this range is based on morphology-based estimates while the lower estimates are based on molecular markers (reviewed in Benoiston *et al.*, 2017). The evolutionary history of diatoms is shaped by endosymbiotic events, of which it has been proposed that there were at least two. The first occurred an estimated 1.5 billion years ago, when a eukaryotic heterotroph assimilated or was invaded by a cyanobacterium (Yoon *et al.*, 2004). Some 500 million years later, a secondary endosymbiosis occurred, in which a eukaryotic heterotroph captured or was invaded by a red alga, establishing the photosynthetic stramenopile lineage to which diatoms belong (Cavalier-Smith, 1999). Recent evidence suggests that the chimeric nature of diatoms is even more complicated, as a range of nucleus-encoded proteins are of green algal origin (Dorrell *et al.*, 2017 and references therein). Diatoms are subclassified into ‘centric’ species, which include two suborders (radial and polar), and ‘pennate’ species, which are further divided into two suborders (raphid and araphid) (Fig. 1). These groups are not monophyletic, as centric diatoms grade into araphid pennates, and araphid pennates grade into the raphe-bearing pennate diatoms, which are a natural group (Alverson and Theriot, 2005).

Diatom cells have a unique structure and features (Fig. 1). Cell sizes are large compared with other oceanic unicellular eukaryotes and are generally in excess of 20 μm in diameter (with maximum cell sizes of 2 mm recorded). Some diatoms are known to form chains. Unlike other organisms, diatom cell membranes are silicified and are referred to as frustules. Frustules are porous, with their nanopores ranging in size from 250 nm to 600 nm; they are generally assumed not to interfere with diatom nutrient uptake (Bhatta *et al.*, 2009). The majority of pennate diatoms contain one central vacuole, while centric species can have many vacuoles. Diatom plastids are surrounded by four membranes, a vestige of the endosymbiosis events that generated the organelle. As with vacuoles, the majority of pennate species have only one plastid, but the number of plastids is variable in the centric species. Nine species of diatoms have had their genomes sequenced: *Thalassiosira pseudonana* (Armbrust *et al.*, 2004), *Phaeodactylum tricornutum* (Bowler *et al.*, 2008), *Thalassiosira oceanica* (Lommer *et al.*, 2012), *Pseudo-nitzschia multistriata* strain B856 (NCBI assembly ASM90066040v1), *Pseudo-nitzschia multiseries* CLN47 (JGI Project ID: 16870), *Synedra acus* (Galachyants *et al.*, 2015), *Fistulifera solaris* (Tanaka *et al.*, 2015), *Fragilariopsis cylindrus* (Mock *et al.*, 2017), and *Plagiostriata* sp. CCMP470 (Sato *et al.*, 2020), while 92 species have available transcriptomes collected through the Marine Microbial Eukaryote Transcriptome Sequencing Project (MMETSP) (Keeling *et al*., 2014). A further seven transcriptomes have been generated by Genoscope and represent seven of the most globally abundant diatom genera not covered by the MMETSP project (Dorrell *et al.*, 2021). The pennate diatom *P. tricornutum* and the centric diatom *T. pseudonana* are laboratory models that can be readily cultured and transformed (Apt *et al.*, 1996; Zaslavskaia *et al.*, 2000; Poulsen *et al.*, 2006; Hopes *et al.*, 2016). Although neither is abundant in the environment, they often harbour genes that have been proposed to contribute to the success of diatoms in the wild.

The ecological success of diatoms is remarkable. They are present in all ocean provinces and are the most abundant photosynthetic producers in the Arctic and the Southern oceans (Malviya *et al.*, 2016). Importantly, they are ruthless competitors for iron, often dominating iron-stimulated blooms (Boyd *et al.*, 2007). This indicates that diatoms are adapted to survive conditions of low iron and are equipped with the molecular machinery necessary to take up and assimilate iron efficiently, without causing damage to their cells through oxidative stress. Rates of iron uptake and use are also linked to nitrogen assimilation and growth: on average, diatoms with higher iron uptake show proportionately greater nitrate uptake and productivity (e.g. Falkowski, 1997). Surprisingly, this is not reflected in their silica uptake, which remains constant under iron supplementation despite faster growth, leading to weakly silicified cells (Boyle, 1998 and references therein). Diatom iron nutrition therefore affects not only metabolism but also the resulting morphology and physical characteristics of diatom cells.

Comparing the cell metal concentrations of diatoms with those of autotrophic flagellates, heterotrophic flagellates, and autotrophic picoplankton in the equatorial Pacific Ocean, Twining *et al.* (2011) reported that phosphorus-normalized Mn, Fe, Ni, and Zn ratios were significantly higher in diatoms than in other cell types. Furthermore, total diatom Fe concentrations per cell exceeded minimum subsistence levels.

Here, we review what is known about iron physiology in diatoms. As far as is possible, we draw comparisons between different diatom species (Sunda and Huntsman, 1995; Strzepek and Harrison, 2004; Kustka *et al.*, 2007). We take a broad perspective that considers how diatoms take up, store, and use iron, in order to shed light on the question of what makes diatoms competitive in handling this micronutrient.

## A portfolio of iron uptake mechanisms shows diatoms are adapted to acquire iron from a variety of sources

The vast majority of iron present in surface ocean seawater is complexed by organic ligands (Gledhill and Buck, 2012). These are classified based on their affinity for iron. The strongest chelates are siderophore molecules, thought to be synthesized largely by bacteria, although fungi, which are of lower abundance in the ocean, are also capable of siderophore synthesis (Renshaw *et al.*, 2002). There are three chemical classes of siderophores—hydroxamate, catecholate, and carboxylate—and their relative contribution to the iron pool of seawater is unknown. Hydroxamate siderophores, which have been detected both in coastal and open ocean waters (Mawji *et al.*, 2008; Boiteau *et al.*, 2016), are hydrophilic and are released by synthesizing species into the water column. In these microorganisms, siderophores are recaptured by specific recognition machinery. It has been proposed that it is these molecules in particular that are available to non-producing species, such as diatoms (Hopkinson and Morel, 2009). Amphiphilic siderophores, which are attached by lipid tails to the cell membranes of producing bacterial species, have received particular attention in the literature on account of their chimeric chemical structures (Martinez *et al.*, 2000; Vraspir *et al.*, 2011). However, they are an unlikely source of iron for non-producers. Other chelates include extracellular polysaccharides and humic acids, which bind iron weakly. Notably, many marine viruses are also capable of chelating iron on their tails and thus may represent a large sink of marine iron (Bonnain *et al.*, 2016). Inorganic mineral iron is periodically available to surface communities, either through aeolian input (i.e. arising from the action of wind) or via upwelling currents that bring remineralized iron from the deep ocean (Mahowald *et al.*, 2008; Boyd and Ellwood, 2010). The most bioavailable and scarcest source of iron is dissolved, uncomplexed iron (Fe′) (Liu and Millero, 2002; Lis *et al.*, 2015).

**Fig. 2.**
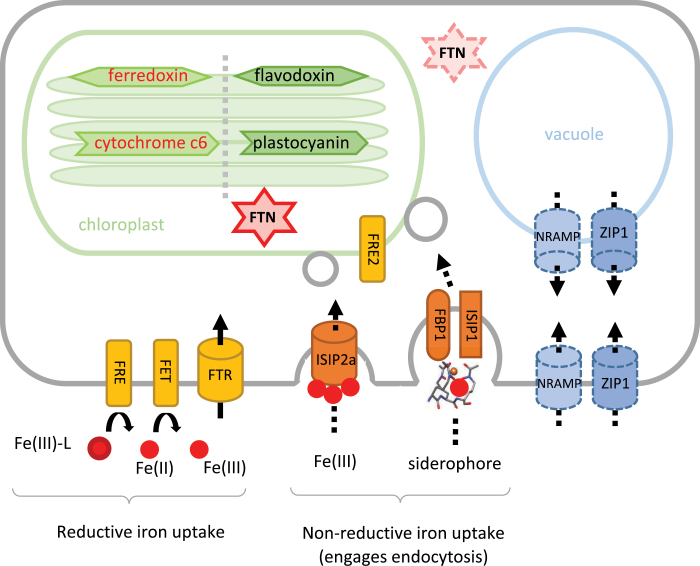
Schematic diagram of iron uptake mechanisms and metabolism in diatoms. A summary schematic of the proteins discussed in this review and of their cellular location. In the chloroplast, we review the replacement of ferredoxin with its iron-free equivalent, flavodoxin, and the substitution of cytochrome *c*_6_ with plastocyanin. Reductive and non-reductive iron uptake mechanisms are considered, the latter employing the cell’s vesicular network to direct iron to the plastid. Two alternative iron storage mechanisms are reviewed: the accumulation of iron in the mineral core of ferritin (FTN), and vacuolar storage. For the storage proteins, the cellular localization of the putative proteins has not been confirmed through microscopy analyses (indicated with dashed lines).

**Fig. 3.**
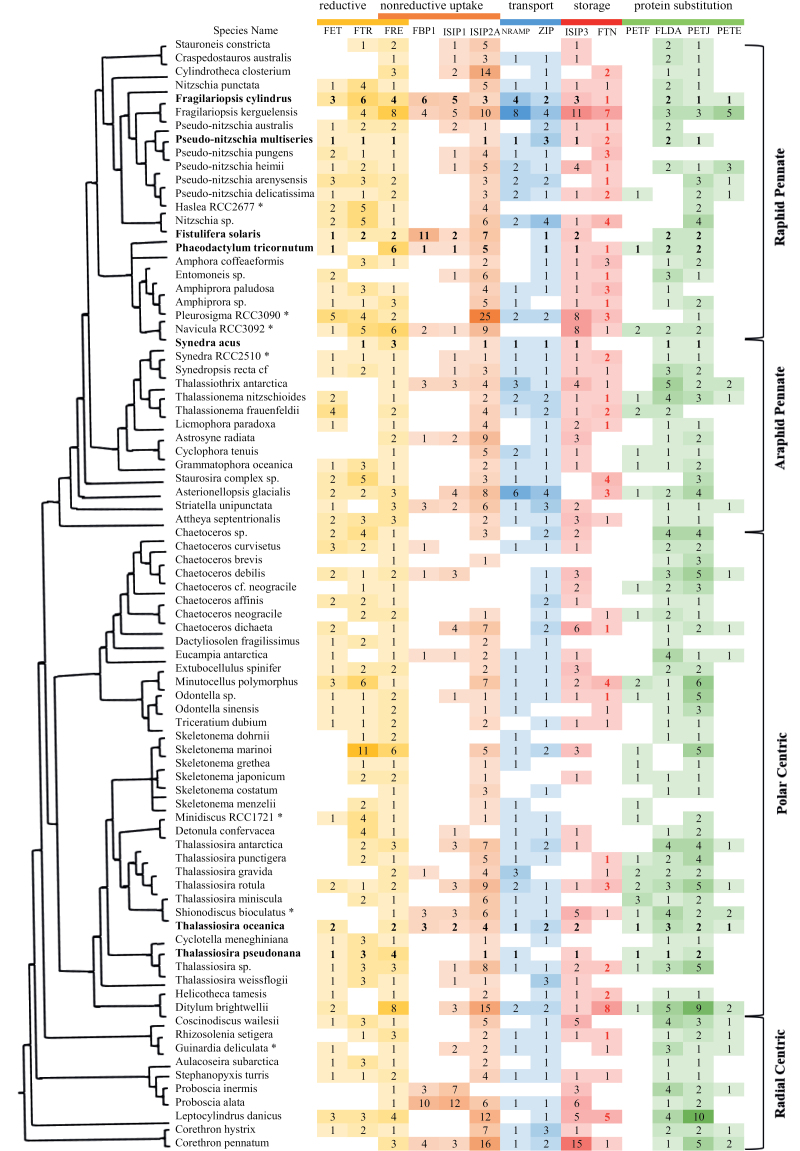
Iron-related genes in diatom transcriptomes. To investigate the evolutionary distribution of iron-related genes, first, representative protein sequences that are well annotated were downloaded from online databases (GenBank, Uniprot, and Ensembl). Further sequences were retrieved from NCBI by BLASTp with a threshold E value of 1×10^–5^ for each gene of interest. Together, these comprised the reference dataset; they were aligned by MUSCLE and transformed into a Hidden Markov Model using HMMER 3.0. The profiles were used for HMMER searches against the diatom transcriptomes from MMETSP alongside seven transcriptomes generated by Genoscope (indicated with asterisks) and diatom genomes (indicated in bold text). In total, our searchable database comprised 82 species, which met decontamination standards described in Johnson *et al.* (2019). The seven species sequenced by Genoscope are among the 10 most abundant diatom genera in the *Tara* Oceans dataset. Where no hits were detected, the grid is blank, otherwise the shade of the colour and the number within indicates the number of distinct transcripts encoding each gene (but not the expression levels). The subcellular localization of FTN was predicted using ASAFind, HECTAR, MitoFates, TargetP, and WolfPSort (Bendtsen *et al.*, 2004; Emanuelsson *et al.*, 2007; Horton *et al.*, 2007; Gschloessl *et al.*, 2008; Fukasawa *et al.*, 2015; Gruber *et al.*, 2015). Sequences predicted to target to the chloroplast are shown in red. It is important to note that this dataset likely misses genes that are under strong transcriptional control, which was not met by the culture conditions during RNA harvesting (e.g. low-Fe-induced genes in diatom cultures grown under Fe-replete conditions), as well as chloroplast-encoded genes. The sequencing depth between the transcriptomes and between genomes varied, and this may have influenced our results. The results presented here may be compared to similar published reviews, notably those by Blaby-Haas and Merchant (2013), Groussman *et al.* (2015), and Behnke and LaRoche (2020). Differences in the numbers of hits retrieved between analyses are likely due to differences in chosen methodologies. FET, ferroxidase; FTR, iron (III) permease; FRE, ferric reductase; FBP, ferrichrome-binding protein; ISIP, iron starvation-induced protein; FTN, ferritin; NRAMP, natural resistance-associated macrophage protein; ZIP, zinc transporter; PETF, ferredoxin; FLDA, flavodoxin; PETJ, cytochrome *c*_6_; PETE, plastocyanin.

Diatoms have a diverse portfolio of iron uptake mechanisms adapted to the sources of iron available in seawater (summarized in Fig. 2). A mechanism whose function has been experimentally verified in the model pennate diatom *P. tricornutum* is the assimilation of uncomplexed iron bound by a transferrin-like protein. The iron starvation-induced protein 2A (ISIP2A), first identified in *P. tricornutum* (Allen *et al.*, 2008), was shown to function as a ‘phytotransferrin’, a protein directed to the cell membrane of cells with carboxylate iron-binding domains, whose iron-chelating properties were pH sensitive (Morrissey *et al.*, 2015; McQuaid *et al.*, 2018). *P. tricornutum* cells deficient in ISIP2A showed reduced Fe′ uptake capabilities, suggesting that this was the primary but not the only mechanism of Fe′ uptake in this species (Kazamia *et al.*, 2018). ISIP2A contains the functional domain PF07692, which was first characterized in the iron-assimilation proteins FEA1 and FEA2 of the green alga *Chlamydomonas reinhardtii* (Allen *et al.*, 2007), and additionally was functionally described in *Dunaliella salina* (Paz *et al.*, 2007). In *C. reinhardtii*, FEA1 and FEA2 were highly expressed under low-iron conditions and facilitated high-affinity iron uptake (Allen *et al.*, 2007), likely in its ferrous form (Narayanan *et al.*, 2011). By contrast, in diatoms, the iron-binding domains of ISIP2A coordinate ferric iron (McQuaid *et al.*, 2018). We searched the available diatom genomes and transcriptomes for genes with a profile that matched a constructed Hidden Markov Model (HMM) for ISIP2A. Our results are presented as a heatmap in Fig. 3, where we show the detection of *ISIP2A*-like genes in 82 diatom species. We retrieved matches for *ISIP2A*-like genes in most diatoms, with some notable exceptions in some species of *Chaetoceros*.

Experimental evidence suggests that colloidal iron, as particulate Fe^2+^, is bioavailable to *P. tricornutum* (Shoenfelt *et al.*, 2017). While aeolian inputs of iron are known to stimulate algal growth, and growth of diatoms in particular, both in short-term ecological experiments (De Baar *et al.*, 2005) and over geological timeframes (Hain *et al.*, 2014; Martínez-García *et al.*, 2014), it has been a long-standing assumption that the iron stimulation is through the increase in Fe′ (Rich and Morel, 1990). Shoenfelt *et al.* (2017) challenged this assumption by demonstrating that the growth rates of *P. tricornutum* cultures improved by glacial dust stimulation beyond what would be expected by the increase in Fe′ alone (the assimilation rates did not match Monod dynamics, and a fit to Michaelis–Menten kinetics resulted in unrealistic half-saturation concentrations). In the same experiments, glaciogenic sediments were more bioavailable to *P. tricornutum* than non-glaciogenic sediments rich in iron, provided cells were in physical contact with the abiotic material. Together, these results implicate an evolved molecular mechanism for the ‘mining’ of mineral iron by some diatoms, which remains to be uncovered. A similar property was observed in the filamentous cyanobacterium *Trichodesmium*, although there too the molecular underpinnings are currently unresolved (Rubin *et al.*, 2011).

There is significant evidence to show that diatoms are able to access the organic pool of ligand-bound iron. Pioneering work by Soria-Dengg and Horstmann (1995) showed that *P. tricornutum* cells were able to access iron from the trihydroxamate siderophores ferrioxamine E (FOE) and ferrioxamine B (FOB), albeit with different kinetics. Working on the same model species, Kazamia *et al.* (2018) demonstrated that this uptake involved endocytosis of the siderophore complex into the cell, with a reduction step to disassociate the bound iron in the vicinity of the chloroplast. Through reverse genetics, the iron starvation-induced protein 1 (ISIP1) was identified as being necessary for the process of endocytosis and siderophore assimilation. Intriguingly, ISIP1 was shown to be largely a diatom-specific protein, implying an evolutionary innovation in this group (Kazamia *et al.*, 2018). The presence of *ISIP1* in diatom genomes and transcriptomes is shown in Fig. 3, although it is important to note that since this gene is highly sensitive to iron status in the cell, it may have been missed from transcriptomes collected from diatoms grown in iron-replete conditions.

Additionally, Coale *et al.* (2019) verified that a previously identified putative siderophore-binding protein, FBP1, which in bacteria binds the hydroxamate siderophore ferrichrome, was indeed required for siderophore assimilation. In our *in silico* searches, 21% of diatom genomes and transcriptomes displayed evidence of a *FBP1* gene (Fig. 3). In physiological experiments on three diatom species reported in Kazamia *et al.* (2018), it was shown that siderophore recognition varied: *P. tricornutum* was unable to use the catecholate siderophore enterobactin as a source of iron, whereas *T. oceanica* assimilated this molecule, but did not respond to ferrioxamine supplementation despite having copies of *FBP1*, *FRE2*, and *ISIP1* in its genome; *T. pseudonana*, which lacks *FBP1* and *ISIP1* genes, was unable to use either siderophore as a source of iron. These observations suggest that while the presence of *FBP1* could be a marker for siderophore uptake in diatoms, further experiments are required to understand what determines substrate specificity in these species.

Laboratory studies on model diatom species, including *P. tricornutum* and *T. oceanica*, report that diatom cells exhibit ferric reductase activity (Maldonado and Price, 2001; Kazamia *et al.*, 2018; Coale *et al.*, 2019). Six genes in *P. tricornutum* have been putatively annotated as ferric reductases, but only two (*FRE1* and *FRE2*) encode two domains indicative of ferric reductase function: the ferric reductase transmembrane component PF01794 and the NAD-binding domain PF08030 (Zhang *et al.*, 2013). The *in silico* prediction for FRE2 is that it is targeted to the chloroplast, albeit with low confidence, whereas the localization of FRE1 is unassigned (Rastogi *et al.*, 2018). We built a HMM for putative diatom ferric reductases based on reference genes from *P. tricornutum*, and searched the available diatom genomes and transcriptomes. Our results show that all species appear to harbour genes encoding ferric reductases, with around two per species on average (Fig. 3). *Fragilariopsis kerguelensis* and *F. cylindrus*, abundant diatoms in the Southern Ocean, stand out because their genomes encode eight and four distinct peptides annotated as ferric reductases, respectively. The coastal species *Ditylum brightwellii* has eight *FRE* genes.

The question arises whether these ferric reductases act on the surface of diatom cells, thus enabling the dissociation of iron from its seawater chelates before assimilation, or act intracellularly. On the one hand, extracellular reduction is unlikely in the highly oxidizing and diffusing environment of seawater. On the other hand, if it were to be coupled to efficient (i.e. rapid and proximal) iron-uptake systems, extracellular reduction could allow cells to dispense with the need to discriminate between iron sources (Shaked *et al.*, 2005). The latter is a system well described in fungi, where ferric iron is displaced from weak ferric ligands by a ferrireductase, then re-oxidized by a multicopper oxidase and finally channelled across the plasma membrane through a Fe^3+^ permease (reviewed by Philpott, 2006). Transcriptional up-regulation of a ferrireductase and two iron permeases was recorded under limiting iron conditions in *T. pseudonana* cells (Kustka *et al.*, 2007). Furthermore, bioinformatic analysis of transcriptome datasets confirm the existence of functional analogues of the yeast-like iron acquisition machinery [the ‘ferrireductase (FRE), multicopper oxidase (FET), iron permease (FTR)’ system] in the model green alga *C. reinhardtii* and across a range of diatom species, including all members of the *Pseudo-nitzschia* and *Fragilariopsis* genera (Fig. 3; also reviewed in Groussman *et al.*, 2015). However, the question of whether this reductive system acts in consort on the surfaces of diatom cells requires experimental verification. Therefore, in Fig. 3 we have indicated the *FRE* genes as acting in both ‘reductive’ and ‘non-reductive’ uptake systems.

## Low iron quota diatoms regulate the amount of iron required for photosynthesis

The metabolic iron requirement (or metabolic iron quota) of diatom cells differs from species to species. This is measured as the Fe:C ratio (μmol Fe mol C^–1^) and is estimated from co-measurements of iron‐limited growth rates and cellular iron content (Sunda and Huntsman, 1995). For example, in *Pseudo-nitzschia* diatoms grown under low-iron conditions, the Fe:C ratio ranged from 2.8 to 3.7 μmol Fe mol C^–1^ (Marchetti *et al.*, 2006). Similar metabolic iron quotas were estimated for *T. oceanica* (at ~2 μmol Fe mol C^–1^), whereas other members of the *Thalassiosira* genus, such as *T. pseudonana* and *T. weissflogii*, had 10-fold higher requirements (Cohen *et al.*, 2018b). In the same study, the metabolic Fe:C ratio for *Corethron hystrix* was estimated at ~40 μmol Fe mol C^–1^ (Cohen *et al.*, 2018b). Diatoms with low metabolic iron quotas are remarkable for their ability to maintain uncompromised photosynthetic function, which is attributed to two main adaptations: (i) preferred use or complete replacement of iron-containing proteins with equivalents that are not dependent on iron, and (ii) re-arrangements of the photosynthetic architecture.

### Diatoms replace iron-requiring proteins with iron-free functional equivalents

**Fig. 4.**
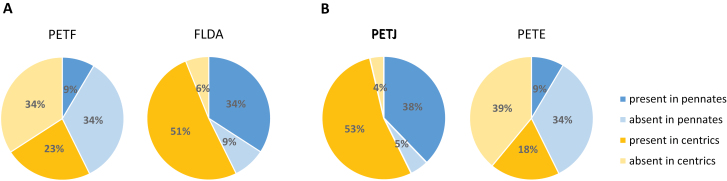
Genes encoding iron-switching proteins in diatom transcriptomes. Both ferredoxin (PETF) and cytochrome *c*_6_ (PETJ) are iron-containing enzymes. As some diatoms are capable of replacing them with the iron-free equivalents flavodoxin (FLDA) and plastocyanin (PETE), respectively, the relative proportions of transcripts encoding these proteins may indicate the dependence of diatoms on these proteins. The pie charts indicate the presence and absence of *PETF* versus *FLDA* (A), and *PETJ* versus *PETE* (B) in pennate and centric diatoms in our database of 82 species. It is important to note that our comparisons here rely on the numbers of genes detected in the transcriptomes of species, and therefore may not be a true reflection of species’ genomes. It is possible that genomes contain genes that were not detected in the transcriptomes, either because the transcriptomes were generated from iron-replete cultures or because species were not sequenced to sufficient depth.

Quantitatively, iron is the most important trace metal in the photosynthetic apparatus because it is involved in the Photosystem II complex (PSII) (which requires two iron atoms per subunit), the cytochrome *b*_6_*f* complex (five iron atoms per monomer), cytochrome *c*_6_ (one iron atom per monomer), the Photosystem I complex (PSI) (12 iron atoms), and ferredoxin (two iron atoms) (Raven *et al.*, 1999; Strzepek *et al.*, 2012). Ferredoxin (PETF) is an iron–sulfur protein that is a key component of the chloroplast electron transport chain. Replacement of ferredoxin by its iso-functional carrier, the flavin-containing protein flavodoxin (FLDA), allows flavin rather than iron to be used for electron transport. The two proteins appear to have similar electrostatic potential profiles, although flavodoxin undergoes two successive single electron reductions, with only the second step matching ferredoxin potential (Sétif, 2001). Among the diatoms with sequence information available, 20 out of 82 species harbour both *PETF* and *FLDA* genes (Fig. 3). There appears to be no phylogenetic relationship between species that encode *PETF*, implying that it was present in the diatom ancestor and that its absence from some species is likely due to gene loss (Figs 3, 4A). The ability to synthesize flavodoxin is widespread among diatoms; we found *FLDA* in 70 out of 82 species (Fig. 3). Since no photosynthetic species are known to survive without either *PETF* or *FLDA*, in transcriptomes where neither transcript was detected, such as that of *Pseudo-nitzschia pungens*, we assume that this was due either to incomplete sequencing or to the stringency of the cut-off parameters chosen for detection in our analysis.

For species that encode both *FLDA* and *PETF*, the ratio of *PETF*:*FLDA* in the cell is controlled by the availability or scarcity of iron. For example, cultures of *F. cylindrus* grown at high iron concentrations produced predominantly ferredoxin, with a small amount of flavodoxin. Ferredoxin was sequentially replaced by flavodoxin in cultures grown with less iron (Pankowski and McMinn, 2009). Similarly, in *P. tricornutum*, *T. oceanica*, and *Pseudo-nitzschia granii*, expression of the *FLDA* gene and the abundance of FLDA protein was much higher under iron limitation (La Roche *et al.*, 1995; Allen *et al.*, 2008; Lommer *et al.*, 2012; Cohen *et al.*, 2018a; Zhao *et al.*, 2018). Under iron-replete conditions, PETF completely replaces FLDA, as observed in natural diatom communities (La Roche *et al.*, 1995; Erdner and Anderson, 1999; McKay *et al.*, 1999; Allen *et al.*, 2008). However, some diatoms contain multiple copies of genes encoding flavodoxin, and only certain isoforms are differentially expressed in relation to iron status. Both *F. cylindrus* and *T. oceanica* have two isoforms of *FLDA*, but transcription of only one isoform is regulated by iron levels (Pankowski and McMinn, 2009; Whitney *et al.*, 2011). Whether possessing a number of flavodoxin isoforms confers an ecological advantage in environments chronically deprived of iron is an interesting question for further investigation.

The strategy of differential transcription of selected genes does not appear to be used by all diatoms. Individual diatom species may be permanently adapted to specific iron regimes in the ocean rather than maintaining transcriptional flexibility. This was supported by analysis of the *Tara* Oceans global dataset for transcriptional regulation of ferredoxin and flavodoxin across a range of algal groups. The dataset combined comprehensive bio-oceanographic genomic and transcriptomic data with iron distributions derived from two global-scale biogeochemical models (Bork *et al.*, 2015; Carradec *et al.*, 2018). Unlike chlorophytes, haptophytes, and pelagophytes, diatoms showed constitutively higher expression of flavodoxin genes than ferredoxin genes, although there was more heterogeneity in expression between species than across these four major groups (Carradec *et al.*, 2018). In *P. granii*, the transcript abundance of *PETF* was appreciably lower than that of *FLDA* regardless of iron status (Cohen *et al.*, 2018a). The diatom *Fragilariopsis curta* appears to have lost *PETF* entirely, and transcription of this species’ remaining *FLDA* gene is not sensitive to iron concentrations (Pankowski and McMinn, 2009). Interestingly, *PETF* is localized in the chloroplast genome in *T. pseudonana* and other diatoms, but has been transferred to the nuclear genome in *T. oceanica*. Compared with its coastal relative *T. pseudonana*, the oceanic diatom *T. oceanica* is highly tolerant to iron limitation. It has been proposed that the transfer of *PETF* from the chloroplast to the nuclear genome might have contributed to the ecological success of *T. oceanica* (Lommer *et al.*, 2010). Whether or not the gene transfer described for *T. oceanica* confers a competitive advantage still needs to be assessed through experimental approaches. That *PETF* is often chloroplast-encoded suggests that analyses of transcriptomes such as the one presented in Fig. 3 may be methodologically biased towards a lack of detection, as RNA harvesting and processing select for nuclear-encoded transcripts.

Cytochrome *c*_6_ acts as an electron carrier between the cytochrome *b*_6_*f* complex and PSI. Cytochrome *c*_6_ (encoded by *PETJ*) may be replaced with the copper-coordinating protein plastocyanin (encoded by *PETE*). The replacement of cytochrome *c*_6_ with plastocyanin is rarer than the replacement of ferredoxin by flavodoxin, and is presumed to have occurred via horizontal gene transfer (Strzepek and Harrison, 2004; Peers and Price, 2006). Only fifteen centric species and eight pennate species show evidence for the presence of a *PETE* gene (Figs 3, 4B). For species that encode both *PETE* and *PETJ*, differences in regulation have been noted. *P. tricornutum* and *F. cylindrus* were found to induce plastocyanin under iron limitation, to temporarily replace cytochrome *c*_6_, and to highly express the gene encoding cytochrome *c*_6_ under iron-replete conditions (La Roche *et al.*, 1996; Cohen *et al.*, 2018a). *F. kerguelensis* is an interesting candidate for investigation because it contains multiple isoforms of plastocyanin (five genes detected using our methods; Fig. 3). In a recent study, Moreno *et al.* (2020) investigated the response of three isoforms of *PETE* to iron in this species, and found that only two were significantly over-represented under low-iron conditions. In *T. oceanica,* which encodes *PETE* together with two functional copies of *PETJ*, the relative expression of plastocyanin was sensitive to iron status, while two genes encoding cytochrome *c*_6_ were weakly but constitutively expressed (Lommer *et al.*, 2012). Similarly, in *P. granii*, transcripts for *PETJ* were weakly abundant regardless of iron status, while *PETE* was highly abundant under iron-replete conditions (Cohen *et al.*, 2018a).

Only a few studies have addressed the question of what the consequences of using plastocyanin are for the copper requirements of cells. For example, the green alga *C. reinhardtii* switches to using cytochrome *c*_6_ instead of plastocyanin only under copper-limiting conditions (Merchant *et al.*, 1991). In *T. oceanica*, copper deficiency inhibited electron transport regardless of iron status, implying that plastocyanin expression was not controlled by iron concentrations (Peers and Price, 2006).

### Low iron quota diatoms lower the ratio of PSI to PSII without compromising photosynthetic output

The PSI complex has the highest iron demand of the light-dependent reactions of photosynthesis. The optimized ratio of PSI: PSII in land plants is approximately 1:1. Comparing the coastal diatom *T. weissflogii* with the open ocean diatom *T. oceanica*, Strzepek and Harrison (2004) found that this ratio was significantly lower in the latter species. This led to the hypothesis that diatoms, in particular those adapted to chronically iron-starved conditions, have streamlined their photosynthetic architecture, minimizing the iron quota necessary for growth. *T. weissflogii* cells contained twice as much PSII than PSI, and *T. oceanica* had reduced PSI demands even further, with a ratio of PSI:PSII of 1:10 (Strzepek and Harrison, 2004). In iron-replete media, the two diatoms grew at comparable rates. However, *T. oceanica* maintained high growth rates (~80% of the rate in iron-replete conditions) in low-iron media that restricted the growth of *T. weissflogii* to ~20% of its iron-replete growth rate. Two studies confirmed similar streamlining in *F. kerguelensis* and *P. granii,* polar diatoms that are abundant in the Southern Ocean and the Arctic Ocean, respectively (Cohen *et al.*, 2018a; Moreno *et al.*, 2020). In *P. granii*, the gene encoding PSI subunit IV (*PSAE*) was almost 4-fold more highly expressed under iron-replete conditions, and protein levels were more abundant by 35-fold compared with their levels under conditions of iron limitation (Cohen *et al.*, 2018a).

Low iron quota diatoms such as *T. oceanica* and *P. granii* are not unique among algae and cyanobacteria in having undergone substitutions and rearrangements in their photosynthetic architecture in order to conserve iron. These traits and plasticity in iron use are widespread (Blaby-Haas and Merchant, 2013; Raven, 2013; Scheiber *et al.*, 2019). However, in an experimental comparison of 29 species of eukaryotic algae and cyanobacteria, Quigg *et al.* (2003, 2011) demonstrated that algae that contain plastids of a secondary endosymbiotic origin, the ‘red plastid’ lineage, which includes the dinoflagellates, haptophytes, and chrysophytes as well as diatoms, had lower stoichiometric quantities of iron per cell compared with the ‘green lineage’ of algae and cyanobacteria. The results from these analyses are difficult to square with measurements of metabolic iron quotas, since the former are carried out under iron-replete conditions. Nevertheless, they suggest that members of the red plastid lineage are capitalizing on more than rearrangements of their photosynthetic architecture for efficient iron use. Studies that look into the adaptations of cell structure, such as the metabolic coupling of plastids and mitochondria (e.g. see some pioneering work by Bailleul *et al.*, 2015), may shed further light on this question.

## Diatoms are able to store iron and regulate its intracellular concentration efficiently

It has been observed that *in situ* iron fertilization, whether artificial or natural, results in blooms dominated by large diatoms, which are often rare in the standing microalgal community (De Baar *et al.*, 2005). These include the chain-forming members of the genera *Fragilariopsis*, *Pseudo-nitzschia*, and *Chaetoceros*, in particular. Comparing their transcriptomes for the analysed genes (Fig. 3), there is no pattern that distinguishes them readily from other diatoms, as traits appear to be species-specific. However, it is important to note that these transcriptomes capture the behaviour of species under laboratory conditions and may not be indicative of their behaviour during a diatom bloom in the wild. The *F. kerguelensis* transcriptome and the *F. cylindrus* genome encode a more comprehensive portfolio of iron-sensitive genes, with multiple isoforms of each gene compared with other species. This is intriguing because, as model polar species, *Fragilariopsis* representatives are considered to be particularly adapted to fluctuating environmental conditions and life in sea ice, which is not ‘iron-limited’ (Mock *et al.*, 2017). Our data suggest that *Fragilariopsis* species are interesting candidates for iron adaptation studies as well.

It is likely that species that respond to iron stimulation by forming blooms are efficient not only at iron uptake, but also in iron homeostasis and long-term storage. One way to assess the capacity for long-term iron storage is to measure the number of divisions that a cell is capable of when ambient iron concentrations drop back to growth-limiting conditions. This can be distinguished from short-term iron homeostasis, which is associated with mechanisms that act on a diurnal scale, where the requirement for iron depends on photosynthetic activity. An alternative is to measure the cell’s metabolic iron quota and subtract this from the total cellular iron carried by a cell under iron-replete conditions. This has been termed the ‘luxury iron’ quota (Sunda and Huntsman, 1997; Marchetti *et al.*, 2009). For example, within the centric *Thalassiosira* genus, the cellular Fe:C ratio of coastal species reached values that were 20–30 times higher than those needed for maximum growth; by contrast, ‘plateau’ iron concentrations in oceanic species were only two to three times higher than maximum needed amounts (Sunda and Huntsman, 1995), indicating that coastal variants had greater capacity to accumulate iron when iron replete.

Two mechanisms have been proposed for the storage of iron inside diatom cells: sequestration into the mineral core of ferritin (FTN) proteins and/or vacuolar storage. FTNs are found in all domains of life, including animals, plants, and microorganisms, and are designed to accommodate large amounts of iron (Theil, 1987). Structurally, FTNs are large protein cages formed by arrays of self-assembling helices with nanocavities (5–8 nm) that catalytically couple iron and oxygen at protein sites for precursors of the cavity mineral. The mineral core of a single protein can store up to 4500 iron atoms (Liu and Theil, 2005). Iron is reversibly released from the core by reduction.

**Fig. 5.**
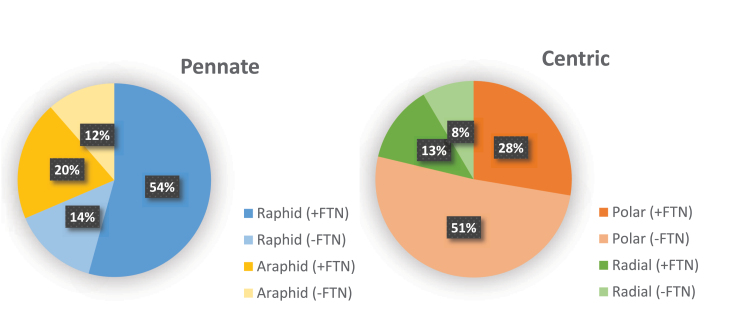
Genes encoding ferritins (FTNs) in diatom transcriptomes. Homologs of *FTN* were retrieved using a HMMER search within available diatom genomes and transcriptomes (see the caption of Fig. 3 for details). Pie charts summarize the presence and absence of *FTN* in raphid versus araphid pennate diatoms, and in polar versus radial centric species. It is important to note that the presence or absence of genes in a transcriptome may not be a true reflection of copy numbers in a genome, either because it was generated from iron-replete cultures or because it was not sequenced to sufficient depth.

We retrieved putative transcripts for *FTN* in diatom transcriptomes and genomes by conducting a HMMER search using the methods described in Fig. 3. We found that ~74% of pennate diatoms contain at least one *FTN* homolog compared with 41% of centric diatoms. Among the centric diatoms the trend for lacking *FTN* skewed towards the polar centric species, of which approximately two thirds were missing the annotation (Fig. 5). The apparent loss of *FTN* in the polar centric diatoms is intriguing and requires further investigation. Looking at the transcriptomes of the three genera of frequently blooming diatoms, *Fragilariopsis* and *Pseudo-nitzschia* species appear to be rich in putative *FTN* genes (Fig. 3). In contrast, our search for *FTN* retrieved hits for only a quarter of the sampled *Chaetoceros* strains (two out of eight). There were notable absences among *Thalassiosira* species (among which five species out of nine do not have a *FTN* gene), including *T. oceanica* and *T. pseudonana*.

In laboratory studies, it was shown that *P. granii,* which harbours a gene encoding *FTN*, was able to undergo several more cell divisions in the absence of iron than the comparably sized oceanic centric diatom *T. oceanica*, which lacks the *FTN* gene, supporting the hypothesis that *FTN* functions in the long-term storage of iron (Marchetti *et al.*, 2009). A corollary of the hypothesis is that *FTN* transcript abundance should increase with iron concentration. However, this has not been universally upheld in laboratory studies. While *P. granii* and *Thalassiosira* sp. NH16 have been observed to increase *FTN* gene expression under high iron concentrations, *Amphora coffeaeformis* exhibited minimal iron-storage capacities and contained two distinct *FTN* genes, one of which increased in expression under iron limitation whereas the second showed no variation with cellular iron status (Cohen *et al.*, 2018b). In fact, we detected three distinct *FTN* transcripts in this species using our methods (Fig. 3). Furthermore, a recent study investigated the community-level response of open ocean plankton ecosystems to iron availability and found that, with the exception of *Pseudo-nitzschia* species, no clear pattern in *FTN* gene abundance or expression and estimated iron levels could be observed, suggesting that iron storage may not be the main function of FTN in most eukaryotic marine phytoplankton (Caputi *et al.*, 2019). Together, these results suggest that FTN in diatoms may have evolved to serve multiple functional roles.

A proposed alternative role for FTN in diatoms is as an iron oxidation enzyme rather than as a long-term iron-storage protein. Working with *P. multiseries*, Pfaffen *et al.* (2013) showed that FTN oxidizes Fe^2+^ at its ferroxidase centres rapidly but forms iron mineral only slowly. In a subsequent study, functional mutagenesis experiments showed that the protein is biochemically optimized for initial Fe^2+^ oxidation but not for mineralization. The authors argued that its primary function therefore might not be in long-term iron storage, but rather in iron homeostasis (Pfaffen *et al.*, 2015). This is the situation in higher plants, where experiments on *Arabidopsis thaliana* demonstrated that *FTN* is regulated by the circadian clock cycle (Duc *et al.*, 2009) and functions to buffer the iron released by degradation from PSI, a protective role in response to photo-oxidative stress (Rossel *et al.*, 2002; Murgia *et al.*, 2007). Similarly, in *C. reinhardtii*, FTN was shown to be required during high ambient iron availability or cellular degradation of iron-containing proteins and protein complexes such as ferredoxin during cellular acclimation to low-iron conditions, indicative of a function in iron homeostasis (La Fontaine *et al.*, 2002; Busch *et al.*, 2008; Long and Merchant, 2008). Finally, in the green picoalga *Ostreococcus tauri*, mutants lacking FTN were less tolerant of low-iron conditions, which induced greater recycling of iron within the cell than in the wild type, further underscoring the importance of this protein in homeostasis. In the same work, the authors proposed that nitrate reductase functioned as an iron-storage protein in this picoalga (Botebol *et al.*, 2015).

**Fig. 6.**
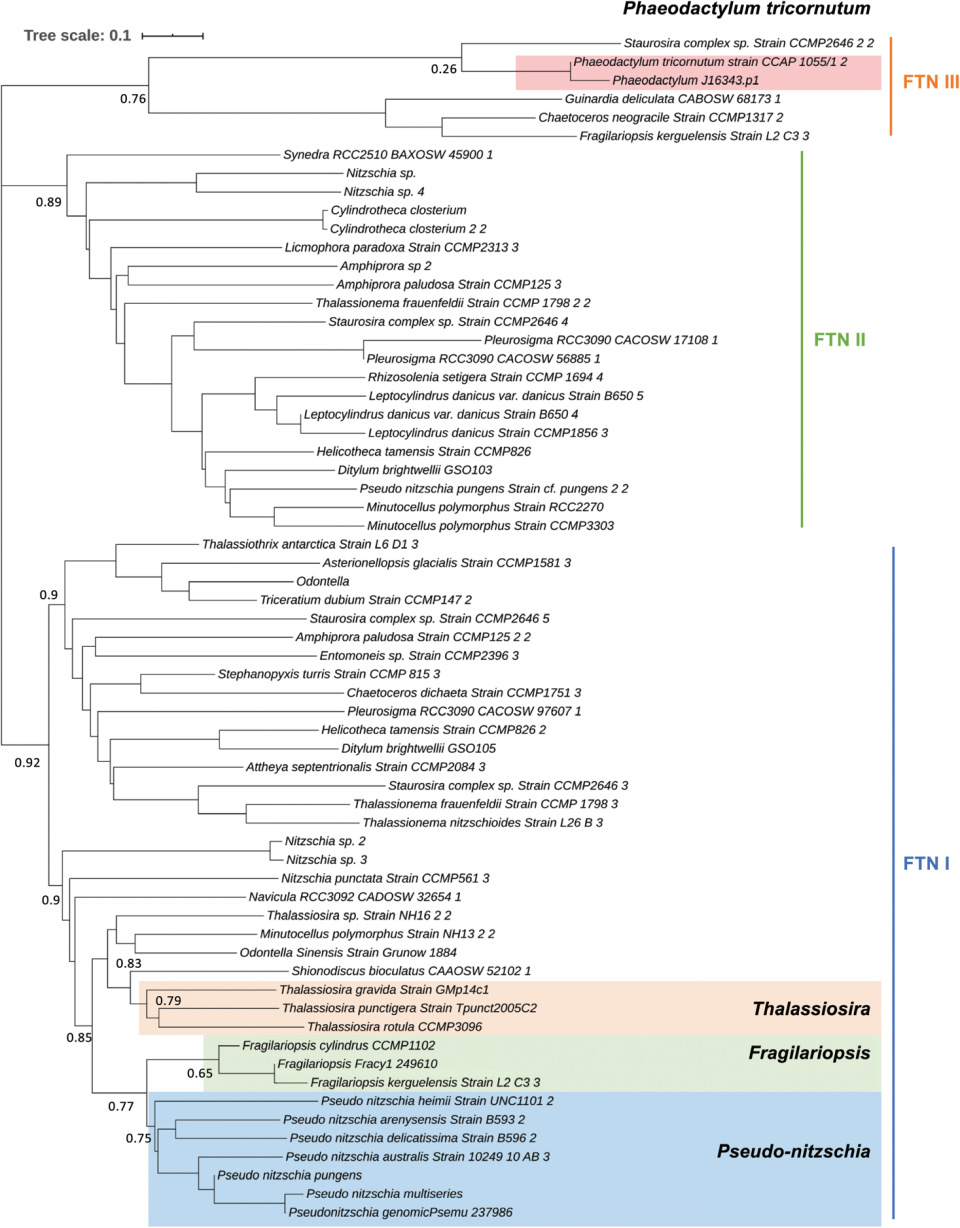
Phylogenetic tree of ferritins (FTNs) in diatoms. The phylogenetic tree was constructed to clarify the evolutionary distance of *FTN* between species. A total of 244 sequences were first retrieved by a HMMER search using the FTN PFAM domain PF00210 as a query in the available 82 diatom transcriptomes/genomes with an E-value cut-off of 1×10^–10^. Retrieved sequences were analysed using the CD-HIT web server and sequences that met a similarity threshold >0.9 were presumed to be duplicates and eliminated. We generated a Hidden Markov Model for *FTN* using *P. tricornutum* and closely aligned sequences retrieved by BLASTp, and this was used as the basis for a second HMMER search of the remaining sequences, with an E-value cut-off of 1×10^–10^, to further reduce the redundancy. This left a total of 64 representative sequences, which captured the diversity of FTNs within the diatoms. Conserved sequences were aligned using the alignment builder in Geneious v.10.2 under default criteria. The tree was drawn with ITOL (https://itol.embl.de/). Numbers beside branches indicate RaxML bootstrap coefficients.

A closer look at diatom FTN phylogeny, which we updated using available diatom transcriptomes (Fig. 6), reveals that the FTNs within the diatoms resolve into several clades. Our phylogeny matches that previously reported by others (e.g. Marchetti *et al.*, 2009; Groussman *et al.*, 2015), but adds more species to the tree. There are two main FTN clades, one of which contains most members of the *Thalassiosira*, *Pseudo-nitzschia*, and *Fragilariopsis* genera, although phylogenetic relationships are not robustly upheld. The second clade contains members of 14 genera, including *Cylindrotheca* and *Leptocylindrus.* The species *Helicotheca tamesis* CCMP826*, Amphiprora paludosa* CCMP125, *Thalassionema fraundfeldii* CCMP1798, *Staurosira* complex sp. CCMP2646, and *Pleurosigma* sp. RCC3090 have annotated FTNs belonging to both clades. To our knowledge, there are no described functional differences between FTNs that belong to the two main groups. Additionally, we found a third group of putative FTNs (which we have annotated as FTN clade III in Fig. 6) with weaker bootstrap support, which includes genes from *P. tricornutum*, *Staurosira* complex sp., *Guinardia deliculata*, *Chaetoceros neogracile*, and *F. kerguelensis*.

**Fig. 7.**
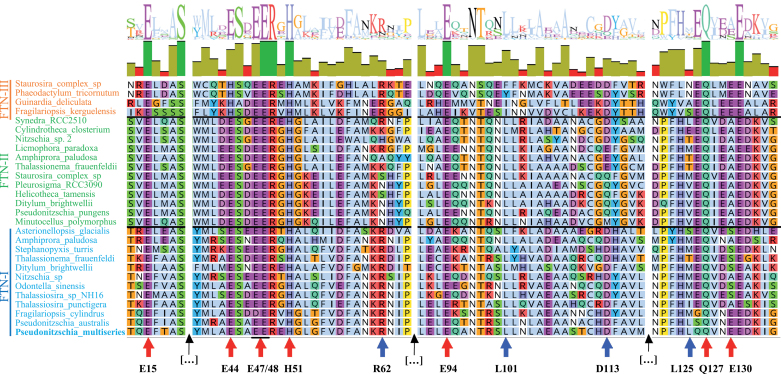
Protein sequence alignment of representative diatom ferritins (FTNs). FTN protein sequence alignment (one-letter code) was carried out using MUSCLE in Geneious v.10.2. At the top is the sequence logo and identity. At the bottom, red arrows highlight the ferroxidase residue sites first identified in *Pseudo-nitzschia multiseries* (Pfaffen *et al.*, 2013). The glutamic acid residues (E15, E47/48, and E94), one glutamine residue (Q127), and the histidine residue H51 are conserved across the three different clades (red arrows). However, the glutamic acid residue E44, which is conserved in FTN I/II, is replaced with histidine in FTN III. These residues are involved in iron binding. Blue arrows show conserved residue pairs essential for iron release in the frog species *Rana catesbeiana* (Jin *et al.*, 2001). The amino acid pairs R62/D113 and L101/L125 are adjacent in three-dimensional space. R62/D113 is conserved in both FTN I and FTN III but not FTN II (which shows significant variation in these positions). Leucine is conserved in position 101 in FTN I and II but not FTN III, while leucine at position 125 is not conserved in any diatoms apart from two *Pseudo-nitzschia* species and *Synedra* sp. RCC2510. The most common replacement for leucine in this position is methionine, as observed in *F. cylindrus* and *Thalassiosira* sp. NH16. Black arrows indicate breaks in the sequence.

We compared the protein sequence of representative FTNs from the three groups by alignment (Fig. 7). We focused on a comparison of iron-binding sites identified by Pfaffen *et al.* (2013) working on *P. multiseries* and key residues identified by Jin *et al.* (2001) working on FTN isolated from the frog *Rana catesbeiana*. It would appear that essential residues involved in iron binding are more strictly conserved in FTN I and II (Fig. 7, red arrows), whereas residues involved in iron release are preserved more across FTN I and III. However, experimental verification is required to validate any implied functional differences.

There is evidence from on-board experiments (which use environmental samples) to suggest that *Pseudo-nitzschia* ferritins serve a long-term storage role in cells, which is not the case in *Thalassiosira* and *Corethron*, in which a role in iron homeostasis is preferred as an explanation (Lampe *et al.*, 2018). Broadly, this is also supported by *Tara* Oceans transcriptomic analyses, which detected up-regulated *FTN* expression in *Pseudo-nitzschia* cells in locations that are rich in iron (Caputi *et al.*, 2019). One structural change that may explain this is the presence of glutamic acid at position 130 in *P. multiseries* FTN, which is absent from other FTN clade I and II species (Pfaffen *et al.*, 2015). It should be noted that E130 is conserved in FTN III diatoms (Fig. 7).

It is likely that a range of proteins that have not yet been explored are involved in iron storage and homeostasis in diatom cells. One notable candidate is the iron starvation-induced protein ISIP3, the most abundant of the three ISIPs in global datasets (Carradec *et al.*, 2018). ISIP3 is typified by a conserved domain of unknown function, DUF305, which belongs to the FTN superfamily (Behnke and LaRoche, 2020). Using our methods, we found transcripts for *ISIP3* across most diatoms, with some notable absences in the coastal *Skeletonema* and *Chaetocerus* genera and an overabundance of putative hits in *Fragilariopsis* (Fig. 3).

An alternative mechanism for long-term iron storage that has been proposed is vacuolar. Iron loading into and release out of the vacuole was first identified in yeast, and was shown to be mediated by natural resistance-associated macrophage proteins (NRAMPs) in *A. thaliana* (Curie *et al.*, 2000; Lanquar *et al.*, 2005). Furthermore, some NRAMP proteins were shown to be functional equivalents of yeast FET proteins, and enabled the transport of iron into the cell (Curie *et al.*, 2000). In *T. pseudonana*, a vacuolar storage mechanism was proposed when it was observed that NRAMP was dramatically regulated by intracellular iron concentrations (Kustka *et al.*, 2007). However, it is important to note that vacuolar localization of NRAMP was proposed but not experimentally validated. In experiments using synchrotron X‐ray fluorescence element mapping, intracellular pockets of iron, indicative of storage features, were identified in both *T. pseudonana* and *T. weissflogii* (Nuester *et al.*, 2012).

A recent study investigated the responses of natural communities, sampled across a gradient of iron concentrations, to on-deck iron stimulation. Metatranscriptome data from incubations was used to assign relative expression of *FTN* and *NRAMP* genes on to three main diatom genera, *Chaetoceros*, *Pseudo-nitzschia*, and *Thalassiosira* (Lampe *et al.*, 2018). The study found that *Pseudo-nitzschia* diatoms were unique utilizers of *FTN* (up-regulating its expression when iron was supplied), whereas the response from *Thalassiosira* species suggested vacuolar storage. In *Chaetoceros*, transcripts of neither *FTN* nor *NRAMP* were abundant, although iron quotas in *Chaetoceros* were often similar to those in *Pseudo-nitzschia* (Lampe *et al.*, 2018). The authors proposed that *Chaetoceros* may have an alternative divalent metal transporter, a protein belonging to the ZIP family, that takes on the function of vacuolar iron transport in these species (Allen *et al.*, 2008). ZIP proteins have been shown to facilitate passive metal transport, including that of ferrous iron, in a range of species (Eide, 2005).

We compared the portfolios of *ZIP*, *NRAMP*, and *FTN* genes across diatoms using all published diatom transcriptomes and genomes, by constructing HMMs for representative genes and scanning the dataset using HMMER (summarized in Fig. 3). This is an amalgamated view, since we did not refine our search for subclasses of NRAMP or ZIP proteins (i.e. the specificity of these genes for iron transport is not confirmed). We found that all diatoms have genes belonging to the *ZIP* family, and that the majority also have *NRAMP* genes (modelled on *F. cylindrus* NRAMP protein). Notable exceptions are most members of the *Chaetoceros* genus, which lack a *NRAMP* annotation. *NRAMP* genes appear to be less abundant among centric species than pennate species, but no robust phylogenetic relationship was observed.

## Synthesis and future directions

There are multiple strands of evidence suggesting that diatoms as a group are successful competitors for iron. Significant research attention has focused on iron uptake and storage, and it is clear that diatoms use a portfolio of strategies to access various forms of iron in the ocean. Remarkably, they have adapted what appear to be ancestrally bacterial uptake proteins to eukaryotic mechanisms. The genes *FBP1* and *ISIP2A* have bacterial origins yet rely on endocytosis to function. Diatom-specific genes have also been observed (e.g. *ISIP1*), implying further innovation in this group to facilitate access to iron. However, experimental verification of the molecular basis of proposed traits remains a major challenge. For example, validation of the *in silico* prediction that FTN is targeted to the chloroplast in diatoms, where iron demands are highest, should be an experimental priority. Additionally, multiple studies have indicated that ZIP and NRAMP proteins, as well as ISIP3, are important for iron physiology, making them clear targets for forward and reverse genetics.

One important avenue for future work is gauging which of these mechanisms is most strongly related to the success of diatoms in the wild. There is evidence that the replacement of ferredoxin with flavodoxin in the photosynthetic machinery of diatom cells is one of the more important adaptations. A recent global survey of gene expression in marine phytoplankton showed that oceanic members of the diatom lineage strongly express flavodoxin over ferredoxin, whereas certain coastal diatoms, which are likely adapted to environments experiencing more frequent and larger fluctuations in iron supply, expressed ferredoxin more highly under iron-replete conditions (Caputi *et al.*, 2019). Of the seven most abundant genera in the ocean (indicated by asterisks in Fig. 3), four contain genes encoding flavodoxin.

Similarly, the expression of ISIPs showed the highest correlation in communities that were sampled from the lowest-iron environments (Kazamia *et al.*, 2018; Caputi *et al.*, 2019). *ISIP1* is notable for being the most sensitive to low-iron status, and likely represents a diatom-specific innovation, although its function requires further elucidation. Its role in mediating siderophore uptake is intriguing, because it suggests a community link between diatoms and siderophore producers. FBP1 is the only siderophore-binding protein that has been described to date, and its distribution appears to correlate with that of ISIP1 in diatom transcriptomes, since most species that encode FBP1 also encode ISIP1, with the exception of *Thalassiosira gravida* and *Chaetoceros curvisetus* (Fig. 3). However, many more species encode ISIP1 than FBP1, so it is possible that ISIP1 has an additional role in cell iron homeostasis or that there are multiple siderophore-binding proteins interacting with ISIP1.

As we garner information on how diatoms put their metabolic portfolios to use in the wild, we will shed a refining light on diatom niches. This will be an important breakthrough in moving away from studies that either focus on individual model species or attempt to draw distinctions between ‘pennates’ and ‘centrics’, or ‘open ocean’ and ‘coastal’ diatoms, while comparing only a few representatives. For the iron uptake, homeostasis, or storage mechanisms that we have reviewed here, there do not appear to be any remarkable patterns that distinguish pennates from centrics. To meaningfully compare open ocean and coastal diatoms, we require more information on the distribution of individual species in the global ocean. The *Tara* Oceans global circumnavigation effort, which catalogued the community composition, metagenomes, and metatranscriptomes of aquatic microorganisms, can offer initial insight. For example, *T. oceanica*, the species most often referred to in the literature as an open ocean species, in fact has a ubiquitous distribution and is frequently found in coastal areas (based on 18S data from the *Tara* Oceans gene catalogue; data not shown). Of the 82 species reviewed here, only *H. tamesis* was not detected within 250 km from the coast during *Tara* Oceans sampling (J. Pierella Karlusich, Institut de Biologie de l’École Normale Supérieure, personal communication), and this species may therefore be considered a true open ocean representative. By contrast, there is a considerable number of coastal species represented in the MMETSP sequence set. The species that were not detected further offshore than 250 km are *Proboscia inermis* (found exclusively in coastal zones at high latitudes), *D. brightwellii*, *Skeletonema marinoi*, *Ticeratium dubium*, *Odontella aurita*, *Navicula* sp., *P. tricornutum*, *A. coffeaeformis*, and *Cylindrotheca closterium*.

Using *Tara* Oceans data, Caputi *et al.* (2019) found that diatom species thrived across a gradient of total iron concentrations and showed remarkable plasticity in their responses to iron availability. The authors concluded that it was not possible to correlate species assemblages to iron levels or transcriptional responses in iron-uptake systems. We believe that further progress will be made when *in situ* studies of species-specific diatom gene expression and analysis of community structure will be coupled to careful characterization of iron sources available in seawater, since it is possible that it is the chemical nature of iron sources that complicates diatom niche separation.
